# Supplementary material for: Deregulation of the Purine Pathway in Pre-Transplant Liver Biopsies Is Associated with Graft Function and Survival after Transplantation
Source: J Clin Med. 2020 Mar 5;9(3):711. doi: 10.3390/jcm9030711 (PMC7141328; doi:10.3390/jcm9030711)
Supplement: Supplementary file 1 [file jcm-09-00711-s001.pdf]

Supplementary materials for:

Deregulation of the purine pathway in pre-transplant liver biopsies associates with graft function and survival after transplantation

Jin Xu, Mohammad Hassan-Ally, Ana María Casas-Ferreira, Tommi Suvitaival, Yun Ma, Hector Vilca-Melendez, Mohamed Rela, Nigel Heaton, Wayel Jassem*, Cristina Legido-Quigley*

*Correspondence and requests for materials should be addressed to:

Cristina Legido-Quigley: cristina.legido.quigley@regionh.dk

Wayel Jassem: wayel.jassem@kcl.ac.uk

Table S1. Misclassification table for the test dataset based on the training dataset model.

True classes	Calculated classes			
		DBD	DCD	Accuracy
	DBD	5	0	100%
	DCD	1	6	85.71%
	Total	12		91.67%
	Fisher's prob.			0.0076

Table S2. Annotation of markers based on molecular weight, retention time and collision induced dissociation fragmentation of 5 metabolites.

Identified as	m/z	Retention time (min)	Observed ion	QC RSD (%)
**Adenine monophosphate**	348.08	17.3	$[M+H]^+$	12.80
**Adenosine**	268.10	14.2	$[M+H]^+$	5.88
**Adenine**	136.06	17.1	$[M+H]^+$	7.16
**Hypoxanthine**	137.05	9.75	$[M+H]^+$	6.07
**Urate**	169.04	11.8	$[M+H]^+$	21.33

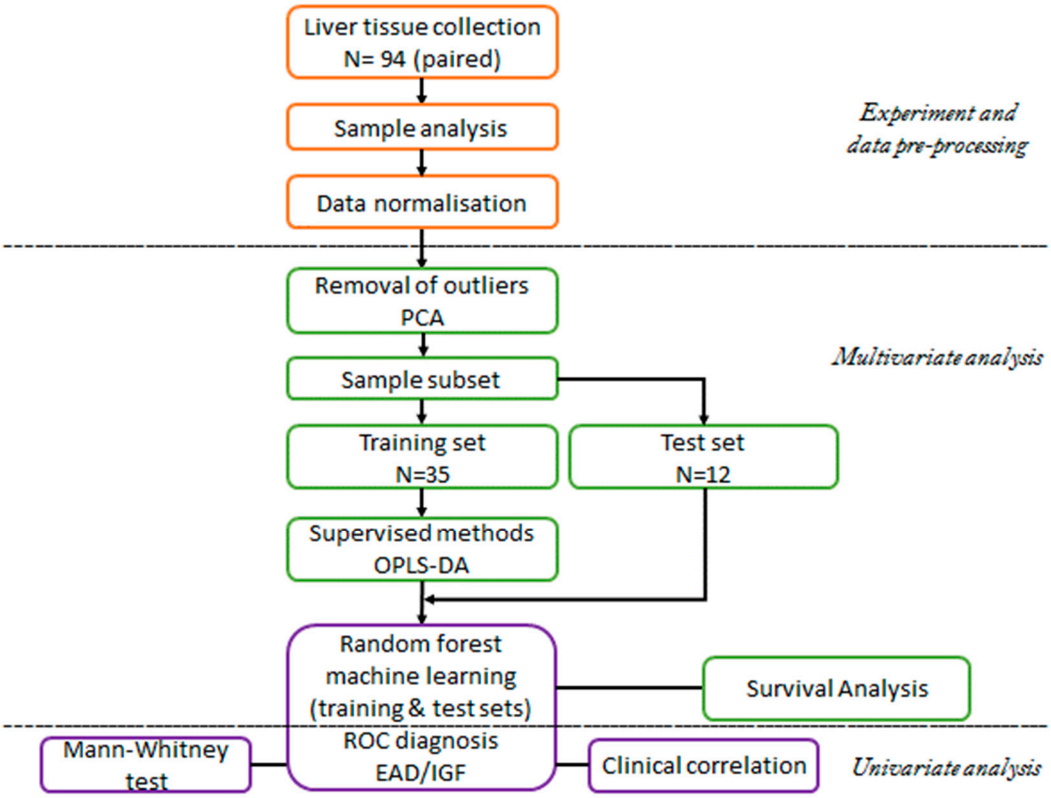

Figure S1. Study workflow.

PCA, principle component analysis; OPLS-DA, orthogonal projections to latent structures-discriminant analysis; EAD; early allograft dysfunction; IGF, immediate graft function; ROC, receiver operating characteristic.

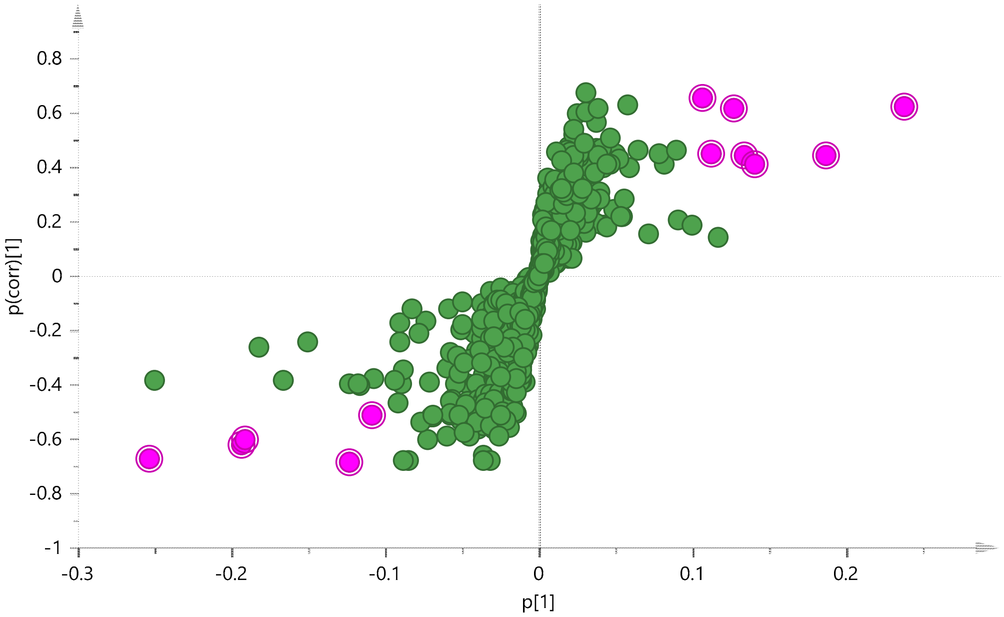

Figure S2. Metabolic feature selection from the S-plot.

All the dots represent detected features, and the pink dots were selected for annotation.

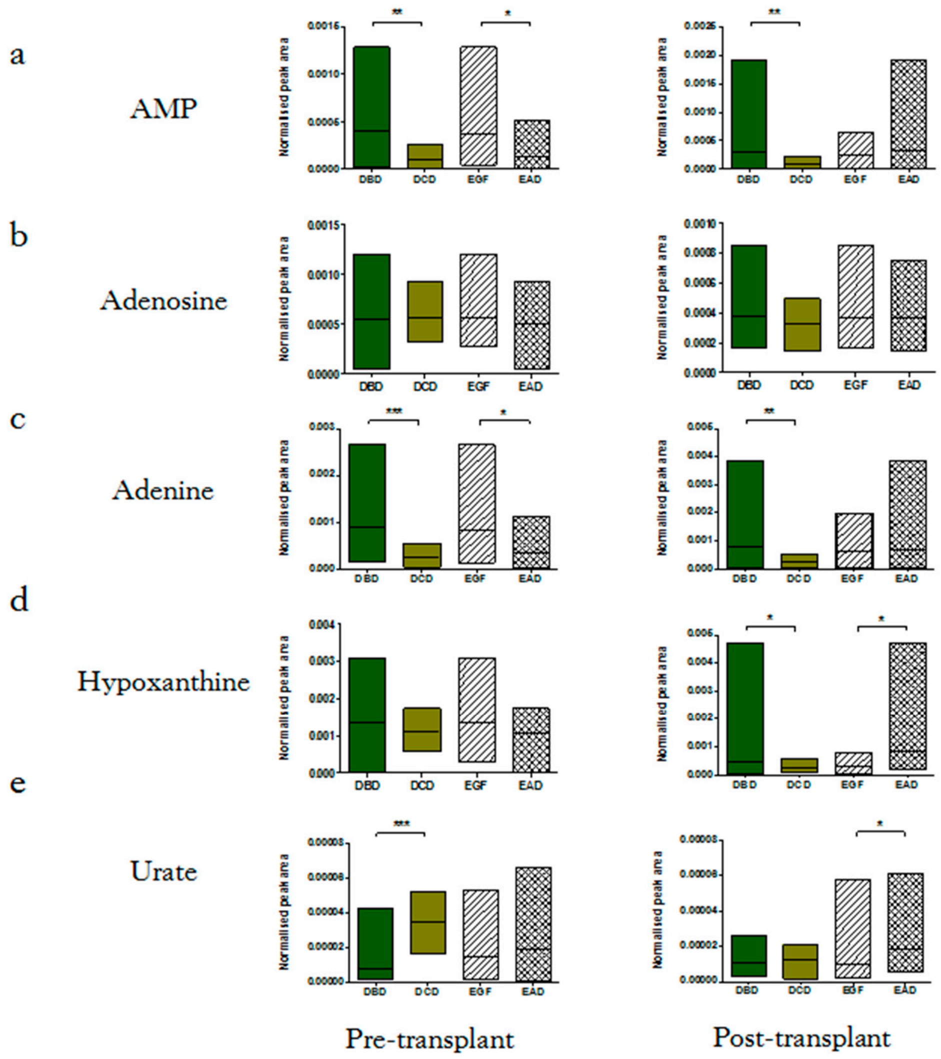

Figure S3. Box plots of 5 metabolites in four groups at both transplant stages. (A) AMP, (B) Adenosine, (C) Adenine, (D) Hypoxanthine and (E) Urate.

AMP, adenosine monophosphate; DBD, donation after brain death; DCD, donation after circulatory death; EGF, early graft function; EAD, early allograft disfunction. Results represented as low-mean-high, p-value was derived from Mann-Whitney test, followed by Benjamini-Hochberg FDR correction (*q<0.05, **q<0.01, ***q<0.001).

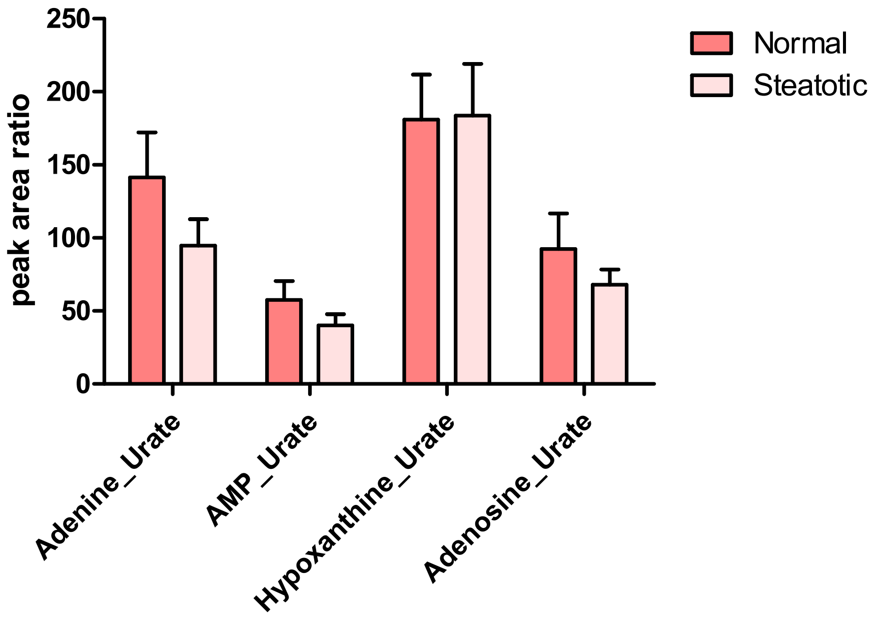

Figure S4. Bar plots of 4 purine ratios in normal (no steatotic, n=21) and steatotic (mild and moderate steatotic, n=26) groups.

AMP, adenosine monophosphate. Results represented as mean with SEM.
